# Draft Genome Sequence of a Vibrio parahaemolyticus Strain, KS17.S5-1, with Multiple Antibiotic Resistance Genes, Which Causes Acute Hepatopancreatic Necrosis Disease in Penaeus monodon in the West Coast of Peninsular Malaysia

**DOI:** 10.1128/MRA.00829-18

**Published:** 2018-07-19

**Authors:** Sridevi Devadas, Subha Bhassu, Tze Chiew Christie Soo, Suhaina Nashath Mohamed Iqbal, Fatimah M. Yusoff, Mohamed Shariff

**Affiliations:** a Selangor Fisheries Biosecurity Centre, Department of Fisheries Malaysia, Sepang, Selangor, Malaysia; b Department of Genetics & Molecular Biology, Institute of Biological Sciences, Faculty of Science, University of Malaya, Kuala Lumpur, Malaysia; c Institute of Bioscience, Universiti Putra Malaysia, Serdang, Selangor, Malaysia; d Department of Veterinary Clinical Studies, Faculty of Veterinary Medicine, Universiti Putra Malaysia, Serdang, Selangor, Malaysia; Georgia Institute of Technology

## Abstract

We report the first draft genome sequence of a Vibrio parahaemolyticus strain (*Vp*_AHPND_), which causes acute hepatopancreatic necrosis disease (AHPND) in Penaeus monodon. The strain has a pVA1-like plasmid carrying *pirA_vp_* and *pirB_vp_* genes. Whole-genome comparisons revealed >98% similarity to *Vp*_AHPND_ isolates from Thailand, Mexico, and Vietnam.

## Abstract (precis)

We report the first draft genome sequence of a Vibrio parahaemolyticus strain (*Vp*_AHPND_), which causes acute hepatopancreatic necrosis disease (AHPND) in Penaeus monodon. The strain has a pVA1-like plasmid carrying *pirA_vp_* and *pirB_vp_* genes.

## ANNOUNCEMENT

Acute hepatopancreatic necrosis disease (AHPND) has caused high mortality in shrimp in Southeast Asia, China, Mexico (1), and Latin America (2). The disease is caused by Vibrio species that contain a virulence plasmid encoding homologs of the Photorhabdus insect-related (Pir) toxin-like genes *pirA_vp_* and *pirB_vp_* (3). In Malaysia, an AHPND outbreak was first reported in 2011 (4); however, there is lack of information about AHPND-causing Vibrio spp. Hence, we present the first draft genome sequence of a Vibrio parahaemolyticus strain (*Vp*_AHPND_), KS17.S5-1, which causes AHPND in Madagascar-Malaysia crossbreed Penaeus monodon. The strain was isolated from the digestive system of moribund shrimp collected from a farm in the west coast of Peninsular Malaysia in January 2017.

The genomic DNA was extracted using the EasyPure bacterial genomic DNA kit (TransGen, China). The paired-end libraries were sequenced using an Illumina MiSeq sequencer and MiSeq reagent kit version 2 (500 cycles) at Majorbio Bio-Pharm Technology Co., Ltd. (Shanghai, China). The clean data pair reads of the strain were 2,344,672 × 2 (average coverage, 219.84×). The genome of the strain was assembled into 79 scaffolds, including 47 large scaffolds (*N*_50_, 405,134 bp) of >1,000 bp, by using SOAP*denovo* v2.04 and GapCloser v1.12 (5). The largest contig was 647,703 bp. The contigs contained 4,928 predicted coding sequences (average length, 907.83 bp), 1 noncoding rRNA gene, and 100 tRNA genes, which were annotated using Glimmer 3.02 (6), Barrnap 0.4.2 (7), and tRNAscan-SE v1.3.1 (8), respectively. The data were searched for homologous sequences using the Basic Local Alignment Search Tool (BLASTn).

The assembled genome of *Vp*_AHPND_ KS17.S5-1 contained an estimated 5,304,178 bp consisting of two chromosomes designated Chr I (∼3,362,757 bp) and Chr II (∼1,873,059 bp), with a G+C content of 45.21%. The strain has a pVA1-like plasmid designated pKS17.S5-1 (∼70 kbp) that carries *pirA_vp_* and *pirB_vp_* genes. Whole-genome comparisons revealed >98% similarity to *Vp*_AHPND_ strains NCKU_TV_3HP, M0605, and 13-028/A3 originating from Thailand (9), Mexico (10), and Vietnam (3), respectively.

The *Vp*_AHPND_ strain KS17.S5-1 contained a thermolabile hemolysin (*tlh*) gene but not thermostable direct hemolysin (*tdh*) and TDH-related hemolysin (*trh*) genes, which are considered important virulence factors of V. parahaemolyticus (11). Nevertheless, the strain contains several pathogenicity-related type II, III, and VI secretion system (T2SS, T3SS, and T6SS), phage-related, multidrug resistance-associated protein (*norM*), vancomycin resistance (*vanZ* and *vanM*), and antibiotic biosynthesis monooxygenase genes. The *norM* gene is able to confer resistance to antibiotics such as norfloxacin, ciprofloxacin, kanamycin, and streptomycin (12), whereas the *vanZ* and *vanM* genes allow high-level resistance to vancomycin (13, 14). This *Vp*_AHPND_ KS17.S5-1 genome information is crucial for the study of the molecular and biological diversity of AHPND strains.

### Data availability.

This whole-genome shotgun project of *Vp*_AHPND_ KS17.S5-1 has been deposited at DDBJ/ENA/GenBank under the accession number PJJY00000000. The version described in this paper is version PJJY01000000. The strain has been deposited at the Universiti Putra Malaysia Institutional Repository (UPMIR).
